# Health technology assessment of long-acting erythropoiesis-stimulating agents for the treatment of renal anemia in China

**DOI:** 10.3389/fphar.2026.1878826

**Published:** 2026-10-02

**Authors:** Xinwen Wang, Huiling Li, Yazhuo Zhang, Yinyin Duan

**Affiliations:** Department of Pharmacy, The Affiliated Huaian No.1 People’s Hospital of Nanjing Medical University, Huaian, Jiangsu, China

**Keywords:** continuous erythropoietin receptor activator, darbepoetin alfa, health technology assessment, long-acting erythropoiesis-stimulating agents, pegmolesatide, renal anemia

## Abstract

**Introduction:**

Renal anemia is a common complication of chronic kidney disease (CKD) and is associated with increased cardiovascular morbidity and mortality and reduced quality of life. Long-acting erythropoiesis-stimulating agents (ESAs) offer advantages in dosing convenience and adherence, but a comprehensive multidimensional evaluation comparing these agents within the Chinese healthcare context is lacking.

**Objective:**

The aim of this study was to conduct a multidimensional quantitative evaluation of long-acting ESAs and provide a reference for drug selection and rational clinical use in medical institutions.

**Methods:**

Based on the method recommended in the Rapid Guideline for Drug Evaluation and Selection in Chinese Medical Institutions (Third Edition), three long-acting ESAs were evaluated in five dimensions, namely, efficacy (28 points), safety (27 points), pharmaceutical properties (27 points), economy (10 points), and other attributes (8 points).

**Results:**

The final assessment scores were 80.39 points for the continuous erythropoietin receptor activator (CERA), 73.04 points for pegmolesatide, and 71.70 points for darbepoetin alfa, in descending order. CERA achieved the highest scores in safety and other attributes, darbepoetin alfa ranked first in economy, and pegmolesatide performed best in pharmaceutical properties.

**Conclusion:**

According to the scoring results, CERA, pegmolesatide, and darbepoetin alfa all exceeded the guideline-defined threshold for strong recommendation (>70 points) and can be included in the list of strongly recommended drugs for medical institutions. This study provides a scientific, reasonable, and safe basis for medical institutions to select long-acting ESAs.

## 1 Introduction

Renal anemia is a common complication of chronic kidney disease (CKD), which severely impairs patients’ quality of life and is closely associated with CKD progression, increased risk of adverse cardiovascular events, and all-cause mortality. Currently, approximately 850 million people worldwide are living with CKD, with a global median prevalence of 9.5% (Bello et al., 2024). Overall, anemia prevalence is 51.5% among non-dialysis CKD patients and over 90% among dialysis patients (Wang et al., 2023). The decreased quality of life in CKD patients is related to their poor prognosis (Toft et al., 2020). Renal anemia can result from multiple factors, including absolute or relative erythropoietin deficiency, disorders of iron metabolism, inhibition of erythropoiesis by uremic toxins, and inflammation (Babitt and Lin, 2012).

Erythropoiesis-stimulating agents (ESAs) are commonly used drugs for the clinical treatment of renal anemia. Currently, clinicians can choose between short- and long-acting ESAs. Short-acting ESAs have a long history of widespread use in China, with numerous domestic and international guidelines and consensus statements supporting their use (Hörl, 2013; Cody and Hodson, 2016). Compared with short-acting ESAs, long-acting ESAs have the advantages of a longer half-life, lower injection frequency, better patient treatment adherence, and reduced workload for medical staff. In recent years, significant progress has been made in clinical research, making long-acting ESAs an important direction for improving the treatment of renal anemia. Long-acting ESAs, including darbepoetin alfa, continuous erythropoietin receptor activator (CERA), and pegmolesatide, have been successively developed and approved for marketing in China, providing new options for the treatment of renal anemia.

Rapid health technology assessment (rHTA) is a process of comprehensively and systematically collecting evidence from clinical research and practice to analyze the safety, efficacy, economy, and patient compliance of drugs used for disease prevention and treatment. It serves as an important technical tool for decision-making in drug supply security. The “Rapid Guide for Drug Evaluation and Selection in Chinese Medical Institutions (Third Edition)” (hereinafter referred to as “the Guideline”) provides a standardized, systematic, and scientific framework for drug evaluation and selection (Zhao et al., 2026). Compared with the Second Edition (Zhao et al., 2023), the Guideline has made key adjustments to the weight distribution in the evaluation system to strengthen the evaluation orientation centered on clinical value. Among the five dimensions, the weights of efficacy and safety have been increased from 27 points and 25 points in the Second Edition to 28 points and 27 points, respectively, while the weight of other attributes has been reduced from 10 points to 8 points, and the weight of pharmaceutical properties has been slightly adjusted to 27 points. On the basis of weight adjustments, the Guideline has deepened and refined the connotation of multiple core indicators, making them more accurate and operable. Its analytical rigor stems from both structural transparency and the systematic application of predefined criteria. For each sub-criterion, scoring was conducted using explicit, evidence-based rules, as outlined in the Selection Guidelines. This framework has been previously applied in rapid comprehensive evaluations of other pharmaceuticals (Lv et al., 2025; Li et al., 2025). Based on the drug evaluation and selection method recommended in the Guideline, we conduct a multidimensional quantitative evaluation of three long-acting ESAs using five dimensions, namely, efficacy, safety, pharmaceutical properties, economy, and other attributes. The aim of this study is to provide a reference basis for medical institutions in selecting and rationally applying such drugs and a basis for their rational clinical use and for developing individualized treatment plans for patients with renal anemia.

## 2 Materials and methods

### 2.1 Evaluation basis

Based on evidence from research and taking China’s national conditions into consideration, the Third Edition Guideline adopts a combination of mini-health technology assessment (Mini-HTA) and the System of Objectified Judgment Analysis (SOJA). The evaluation criteria and corresponding weighting coefficients were developed through a modified Delphi consensus process. Therefore, based on the evaluation and selection method recommended in the Guideline, drugs were quantitatively scored and comprehensively evaluated in five dimensions: efficacy (indications, guideline recommendations, and clinical efficacy), safety (adverse reactions, drug interactions, and medication use in special populations), pharmaceutical properties (pharmacological effects, *in vivo* processes, and pharmacy and storage conditions), economy (comparison of average daily costs of drugs with the same generic name and alternative drugs), and other attributes (national medical insurance, national essential drugs, etc.). The full scoring matrix, including detailed evaluation criteria, evidence sources, and rationales for each score, is provided in Supplementary Table S1.

### 2.2 Evaluation of drugs

Long-acting ESAs are defined as erythropoiesis-stimulating agents with an elimination half-life sufficient to permit a dosing interval of at least once weekly, including darbepoetin alfa, CERA, and pegmolesatide. As of 31 March 2026, these three agents represent all long-acting ESAs approved by the National Medical Products Administration (NMPA) for the treatment of renal anemia. Of note, pegmolesatide is structurally distinct from peginesatide (formerly marketed as Omontys), an earlier peptide-based ESA that was voluntarily withdrawn worldwide in 2013 due to serious hypersensitivity reactions; no peginesatide-derived data were used in the present evaluation.

Inclusion criteria: (1) approved by the NMPA for renal anemia in patients with CKD; (2) meet the definition of a long-acting ESA; and (3) publicly available package insert/clinical trial data/pricing information.

Exclusion criteria: (1) short-acting ESAs; (2) agents without an approved indication for renal anemia; and (3) non-ESA agents, such as hypoxia-inducible factor prolyl hydroxylase inhibitors (HIF-PHIs).

Detailed information on the three long-acting ESAs is presented in Table 1.

**TABLE 1:** Drugs included in the evaluation.

Drug name	Brand name	Specification	Manufacturer	Dosage form (route of administration)	Approved regions (year)
Darbepoetin alfa	NESP (Novel Erythropoiesis Stimulating Protein)	10 μg/0.5 mL 20 μg/0.5 mL 30 μg/0.5 mL 40 μg/0.5 mL 60 μg/0.5 mL	Kyowa Kirin Co., Ltd. Terumo	Injection (intravenous/subcutaneous)	China (2020), United States (2001), Europe (2007), and Japan (2007)
CERA	MIRCERA	50 μg/0.3 mL 75 μg/0.3 mL 100 μg/0.3 mL 120 μg/0.3 mL 150 μg/0.3 mL	Roche Pharma (Schweiz) AG	Injection (intravenous/subcutaneous)	China (2018), United States (2007), Europe (2007), and Japan (2008)
Pegmolesatide	Shengluolai	2.0 mg/0.5 mL 4.0 mg/1 mL 6.0 mg/1.5 mL	Jiangsu Hansoh Pharmaceutical Group Co., Ltd.	Injection (subcutaneous)	China (2023)

### 2.3 Retrieval and evaluation of relevant evidence

Drug labels and drug registration information were consulted. Databases including PubMed, Embase, the Cochrane Library, Web of Science, the China National Knowledge Infrastructure (CNKI), the WanFang Database, and the Chinese Biomedical Literature Database (CBM) were searched electronically to collect the latest evidence related to long-acting ESAs, such as diagnosis and treatment norms, clinical guidelines, expert consensus statements, systematic reviews/meta-analyses, and randomized controlled trials (RCTs). The search time frame spanned from database inception to 31 March 2026. The PubMed search strategy is detailed in Supplementary Table S2. Adverse drug reactions were graded primarily based on drug labels and the Common Terminology Criteria for Adverse Events (CTCAE) Version 6.0. Drug prices were obtained from authoritative information query platforms, including the latest listed prices on provincial procurement platforms, the National Health Insurance Information Database, and the Center for Drug Evaluation of the National Medical Products Administration (NMPA). This assessment covered multiple dimensions, criteria, weights, and implications related to drug evaluation and selection, with quantitative scoring of drugs conducted in five dimensions: efficacy (28 points), safety (27 points), pharmaceutical properties (27 points), economy (10 points), and other attributes (8 points). This framework emphasizes a clinical value-centered approach to drug evaluation and supports medical institutions in the objective selection and evaluation of drugs.

The inclusion criteria were as follows. (1) Study type and language: for efficacy and safety assessment, randomized controlled trials and systematic reviews/meta-analyses were included; for guideline recommendation assessment, domestic and international clinical guidelines and expert consensus statements were included; for pharmaceutical properties assessment, drug labels and drug registration information were included. The literature language was restricted to Chinese and English. (2) Study population: Patients with chronic kidney disease complicated by renal anemia, including both non-dialysis-dependent and dialysis-dependent patients. (3) Intervention and comparator: Studies comparing darbepoetin alfa, CERA, or pegmolesatide with placebo, short-acting ESAs, or other active comparators. (4) Efficacy outcomes: The primary efficacy endpoint was hemoglobin (Hb) level. Secondary efficacy endpoints included Hb values at various time points and their changes from baseline, time to Hb response, and transfusion requirements. (5) Safety outcomes: Adverse events, serious adverse events, and all-cause mortality.

The exclusion criteria were as follows: (1) study design: narrative/traditional reviews (excluding systematic reviews/meta-analyses), case reports, case series, retrospective studies, editorials, and letters. (2) Duplicate publications: duplicate publications of the same study data; only the most informative publication was included. (3) Non-target population: studies in populations with non-renal anemia, such as chemotherapy-induced anemia, myelodysplastic syndromes, or pure red cell aplasia. (4) Conference abstracts: conference abstracts without available full text. (5) Unavailable data: studies with incomplete data from which valid data could not be extracted.

Two pharmacists independently conducted the evaluation. When there was a significant discrepancy in the evaluation results between the two pharmacists (>3 points), relevant experts in the field were invited to discuss the discrepancy and reach a final decision. The evaluation results were ultimately used in drug selection by medical institutions and decision-making regarding clinical medication regimens. A 100-point scale was adopted for quantitative scoring, and the scoring results served two purposes: (1) for the introduction of new drugs: a score of >70 points is recommended as strongly recommended; a score of 60–70 points is recommended as weakly recommended depending on the availability of alternative therapeutic drugs in clinical practice; a score of <60 points is recommended as not recommended. (2) For the withdrawal of drugs: a score of <60 points is recommended for withdrawal; a score of 60–70 points is recommended for temporary retention or withdrawal depending on the availability of alternative therapeutic drugs in clinical practice; a score of >70 points is recommended for retention.

#### 2.3.1 Inter-rater agreement

Preliminary independent ratings were performed by two clinical pharmacists. Inter-rater consistency for all domain-level items was assessed using the intraclass correlation coefficient (ICC). The ICC was calculated using a two-way random-effects model for absolute agreement (ICC[2,1]) in SPSSAU software (version 26.0), with the analytical unit being the 20 domain-level rating items across the three drugs. The final ICC was 0.984 (95% CI: 0.976–0.989), indicating excellent inter-rater agreement.

## 3 Results

### 3.1 Efficacy (28 points)

#### 3.1.1 Indications

##### 3.1.1.1 Disease severity

Anemia is a common clinical manifestation in patients with kidney disease, serving as both a significant complication and a frequent comorbidity. Renal anemia impairs the quality of life of patients with kidney disease and increases the risk of disease progression, end-stage renal disease, cardiovascular events, and mortality (Eriksson et al., 2016; Hoshino et al., 2020). It is classified as a high-risk disease, and its progression may be life-threatening or cause permanent damage. The three long-acting ESAs are routine therapeutic drugs for this condition, used to correct anemia, improve prognosis, and support long-term rehabilitation. Each received a score of two points.

##### 3.1.1.2 VEN analysis

ESAs are first-line therapeutic drugs for anemia in CKD. Their core advantages lie in extensive clinical experience and clear efficacy data. In patients with renal anemia, after addressing the correctable causes of anemia, ESAs are recommended as first-line treatment (Kidney Disease: Improving Global Outcomes KDIGO Anemia Work Group, 2026). Darbepoetin alfa, CERA, and pegmolesatide all scored five points.

#### 3.1.2 Guideline recommendations

Darbepoetin alfa, CERA, and pegmolesatide are recommended in multiple guidelines (Kidney Disease: Improving Global Outcomes KDIGO Anemia Work Group, 2026; Renal Anemia Guidelines Working Group, 2021; Consensus Expert Group and Dialysis Professional Committee of the Chinese Non-Public Hospital Association, 2024; Clinical practice guidelines for management of chronic kidney disease during peridialysis in China, 2025 Working Group of the Kidney Disease Dialysis Special Committee of the China Association of Non-Public Medical Institutions, 2025; Bhandari et al., 2025). Darbepoetin alfa and CERA both received the highest recommendation grade of IA, with a guideline recommendation score of 10 points each. As a newly marketed drug with limited research on safety and efficacy, pegmolesatide is less commonly used in clinical practice. Due to its recent market entry and current availability limited to China, it is recommended only in Chinese guidelines and consensus statements and has not yet been included in most international guidelines. Therefore, its score was adjusted to nine points.

Recommendations from domestic and international guidelines or consensus statements are shown in Table 2.

**TABLE 2:** Recommendations from national and international guidelines/consensus.

Name of the guidelines	Guide developers and sources	Name of drug	Recommended content	Level of evidence
Clinical practice guidelines for the diagnosis and treatment of renal anemia in China (Renal Anemia Guidelines Working Group et al., 2021)	Renal Anemia Guidelines Working Group, Chinese Society of Nephrology Physicians, and Chinese Medical Doctor Association	Darbepoetin alfa CERA	ESAs are effective for the treatment of renal anemia. ESAs significantly reduce transfusion frequency and alleviate anemia-related symptoms in patients with CKD. The selection of ESA type and the decision regarding the initial ESA treatment dose should be based on the Hb level and clinical status of CKD patients	1A
Chinese expert consensus on long-acting erythropoiesis-stimulating agents for the treatment of renal anemia (2024 Edition) (Consensus Expert Group and Dialysis Professional Committee of the Chinese Non-Public Hospital Association, 2024)	Consensus Expert Group and Dialysis Professional Committee of the Chinese Non-Public Hospital Association	Darbepoetin alfa CERA Pegmolesatide	Long-acting ESAs are recommended for the treatment of renal anemia in non-dialysis-dependent CKD patients. Long-acting ESAs are recommended for the treatment of renal anemia in dialysis-dependent CKD patients It is suggested that long-acting ESAs may be used for the treatment of renal anemia in patients with low response to short-acting ESAs. For treatment-naive patients receiving ESAs, it is recommended that the initial dose of darbepoetin alfa can be administered as a fixed dose (20 µg) or calculated based on body weight (0.45 μg/kg), once weekly, via subcutaneous or intravenous injection; the initial dose of CERA is 0.6 μg/kg, once every 2 weeks, via subcutaneous or intravenous injection; the initial dose of pegmolesatide is 0.04 mg/kg, once every 4 weeks, via subcutaneous injection	1A
Clinical Management Guidelines for Delaying the Progression of Chronic Kidney Disease (2025 Edition) (Expert Group of Clinical Management Guidelines for Delaying the Progression of Chronic Kidney Disease, 2025)	Expert Group of Clinical Management Guidelines for Delaying the Progression of Chronic Kidney Disease (2025 Edition)	Darbepoetin alfa CERA Pegmolesatide	For CKD patients with HGB ≤100 g/L, it is recommended to initiate ESAs or hypoxia-inducible factor prolyl hydroxylase inhibitors (HIF-PHIs) for the treatment of renal anemia. For anemic patients with HGB >100 g/L, individualized treatment with ESAs or HIF-PHIs may be considered when there is a need to increase HGB levels to improve the quality of life. The efficacy of the pegmolesatide group in increasing HGB levels was non-inferior to that of the short-acting erythropoietin (EPO) group. Compared with short-acting ESAs, long-acting ESAs have a longer half-life, with potential medication advantages of reducing injection frequency, decreasing the workload of medical staff, and improving patients’ treatment adherence	A
Clinical Practice Guidelines for the Management of Peri-Dialysis Chronic Kidney Disease in China (2025 Edition) (Clinical practice guidelines for management of chronic kidney disease during peridialysis in China, 2025 Working Group of the Kidney Disease Dialysis Special Committee of the China Association of Non-Public Medical Institutions, 2025)	Nephrology and Dialysis Professional Committee of Chinese Non-Public Medical Institutions Association	Darbepoetin alfa CERA Pegmolesatide	It is recommended that for peri-dialysis CKD patients with anemia, non-renal causes of anemia should be corrected before initiating drug treatment, and then, therapeutic drugs should be selected individually based on the patient’s clinical manifestations and laboratory test indicators.​ Therapeutic drugs for renal anemia include iron preparations, ESAs, and HIF-PHI.	1A
KDIGO 2026 Clinical Practice Guideline for the Management of Anemia in Chronic Kidney Disease (CKD) (Kidney Disease: Improving Global Outcomes KDIGO Anemia Work Group, 2026)	Kidney Disease: Improving Global Outcomes (KDIGO) Anemia Work Group	Darbepoetin alfa CERA	In patients with anemia and CKD in whom correctable causes of anemia have been addressed, we suggest using an ESA rather than a HIF-PHI as first-line treatment of anemia In patients with anemia and CKD G5D receiving HD or peritoneal dialysis, we suggest initiation of ESA therapy when the Hb concentration is ≤ 9.0–10.0 g/dL (≤90–100 g/L)	2D
UK kidney association clinical practice guideline: update of anemia and chronic kidney disease (Bhandari et al., 2025)	Bhandari S	Darbepoetin alfa CERA	ESAs should be administered to anemic patients with CKD to improve quality of life and physical function and to avoid blood transfusions, particularly in transplant-eligible patients	1B

#### 3.1.3 Clinical efficacy

This evaluation applies to adult patients with renal anemia. Age, comorbidities (diabetes, cardiovascular disease, and chronic inflammation), dialysis status, and residual renal function may affect individual ESA responsiveness. All efficacy evaluations assume adequate iron stores as iron deficiency is the most common cause of ESA hyporesponsiveness, and we note that iron status should be assessed and corrected before and during ESA therapy. Individual dose titration based on Hb response, starting from the initial dosing regimen specified in the prescribing information, is standard clinical practice. Based on the literature analysis and clinical practice, the primary efficacy endpoint was Hb level. Secondary efficacy endpoints included Hb values at various time points and their changes from baseline, time to Hb response, and transfusion requirements.

A randomized, double-blind study showed that Hb levels increased significantly 1 month after treatment with darbepoetin alfa, reaching 125 g/L at 3 months, a level that was significantly higher than that in the placebo group (*p* < 0.001). The TREAT trial demonstrated that darbepoetin alfa significantly reduced transfusion requirements (HR 0.68, *p* < 0.001) but did not reduce the primary composite cardiovascular/renal endpoint (HR 1.05, *p* = 0.41) and was associated with an increased risk of stroke (HR 1.92, *p* < 0.001) (Pfeffer et al., 2009). In a randomized, open-label study on 139 hemodialysis patients, Bernieh et al. (2014) compared darbepoetin alfa, administered once weekly or once every 2 weeks, with short-acting epoetin administered two to three times weekly. Darbepoetin alfa achieved target Hb more effectively (a higher proportion of Hb measurements within the target range), required significantly fewer dose adjustments, and was associated with a significantly lower incidence of vascular access thrombosis.

A clinical trial conducted in China evaluated the efficacy and safety of CERA in improving anemia in dialysis-dependent CKD patients. During the assessment period (17–24 weeks), the Hb response rate in the CERA treatment group was 87.12%, with an average increase in Hb levels of 28.0 g/L from baseline, while the Hb response rate in the short-acting ESA group was 87.22%, with an average increase of 32.7 g/L from baseline. There was no statistically significant difference between the two groups, indicating that CERA is non-inferior to short-acting ESAs in correcting anemia in dialysis-dependent CKD patients and has good safety and tolerability (Qian et al., 2012). A meta-analysis including six RCTs involving 1,626 dialysis-dependent CKD patients showed no statistically significant differences in Hb levels at the end of treatment or changes in Hb levels from baseline between CERA and short-acting ESAs (Saglimbene et al., 2017). Another meta-analysis incorporating 13 Phase III trials with 2,060 hemodialysis CKD patients further confirmed that CERA, short-acting ESAs, and darbepoetin alfa have comparable efficacy in improving Hb levels and similar safety profiles in hemodialysis patients (Locatelli et al., 2016).

A Chinese clinical study showed that during the evaluation period, the mean changes from baseline for pegmolesatide and short-acting ESAs were 0.7 g/L and −2.2 g/L, respectively. The between-group difference was 2.9 g/L (95% CI = 1.1–4.7, *p* = 0.002). Pegmolesatide was superior to short-acting ESAs in improving and maintaining Hb levels in dialysis patients, with no safety concerns identified (Zhang et al., 2023).

The three long-acting ESA-related RCTs primarily included in this study exhibited relatively good evidence quality. Among them, the darbepoetin alfa study conducted by Pfeffer et al. (2009) was a large-sample, multicenter, double-blind, placebo-controlled clinical trial with robust random sequence generation, allocation concealment, blinding implementation, and completeness of outcome data, resulting in an overall low risk of bias and a rating of high-quality evidence according to the GRADE criteria. Both the CERA study by Qian et al. (2012) and the pegmolesatide study for anemia in dialysis patients by Zhang et al. (2023) were multicenter, open-label, active-controlled clinical trials. These two studies employed standardized randomization protocols, had complete outcome data, and showed no evident selective reporting bias; however, they were limited by the absence of blinding, introducing a degree of subjective assessment bias. Specifically, the former had an elevated overall risk of bias due to inadequate reporting of the allocation concealment method, leading to a downgrade to moderate-quality evidence, whereas the latter was rated as providing moderate-quality evidence under the GRADE framework because it exclusively enrolled Chinese dialysis patients, which restricted population representativeness.

Compared with short-acting ESAs, long-acting ESAs have a longer half-life and offer potential clinical advantages, including less frequent injections, reduced workload for medical staff, and improved patient treatment adherence. In this study, evaluations were conducted based on consistent identification by pharmacists and verification using the Delphi method. Scores were assigned based on whether the primary and secondary efficacy endpoints were superior, non-inferior, or showed no statistically significant difference. Superiority in the primary efficacy endpoint was assigned 5 points, whereas superiority in the secondary efficacy endpoint was assigned 3 points. Non-inferiority or no statistical difference in the primary and secondary efficacy endpoints was assigned 4 points and 2 points, respectively.

In summary, for the primary endpoints, darbepoetin alfa, CERA, and pegmolesatide scored 5, 4, and 5 points, respectively. For the secondary endpoints, they scored 3, 2, and 3 points, respectively. All three agents met clinical efficacy criteria and were awarded 2 points each.

The efficacy score results are shown in Table 3.

**TABLE 3:** Efficacy scoring results.

Efficacy (28 points)		Grading criteria	Darbepoetin alfa	CERA	Pegmolesatide
Indications (8 points)	Disease severity	—	—	—	—
	Very high risk. The disease may be life-threatening at any time	3	—	—	—
	High risk. Disease progression may be life-threatening or cause permanent impairment	2	2	2	2
	Low risk. The disease is self-limiting or progresses slowly, with little impact on the patient’s life expectancy	1	—	—	—
	VEN (5)	—	—	—	—
	Clinically necessary, vital drugs	5	5	5	5
	Clinical need, essential drugs	3	—	—	—
	More medicines available, nonessential drugs	1	—	—	—
Guide recommendation (10 points)	Strong recommendation: Diagnostic and treatment criteria, consensus statements/administrative measures issued by national health administrative departments, guidelines with Level I recommendation (A-level evidence: 10, B-level evidence: 9, C-level evidence: 8, and others: 7)	10	10	10	9
	Weak recommendation (A-level evidence: 6, B-level evidence: 5, and C-level evidence and others: 4)	6	—	—	—
	Expert consensus recommendation (consensus issued by national-level academic societies)	3	—	—	—
Clinical efficacy (10 points)	The primary efficacy endpoint indicators	5	5	4	5
	The secondary efficacy endpoint indicators	3	3	2	3
	Can provide clinical efficacy data	2	2	2	2
Effectiveness score		—	27	25	26

### 3.2 Safety (27 points)

#### 3.2.1 Adverse reactions

The severity of adverse reactions was assessed using the CTCAE (Version 6.0) grading system (NCI, 2025).

For darbepoetin alfa, mild-to-moderate adverse reactions included hypertension, hypersensitivity reactions (≥10%), rash/erythema, and injection site pain (≥1% to <10%), scoring 1 point. Severe adverse reactions included stroke (≥1% to <10%), convulsions, and thromboembolic events (≥0.1% to <1%), scoring 2 points.

The most common adverse reactions of CERA were hypertension (≥1% to <10%), vascular access thrombosis (≥0.1% to <10%), and headache (≥0.1% to <10%), scoring 2 points. Serious adverse reactions were rare, including allergic reactions, hypertensive encephalopathy, and severe maculopapular rash (<0.01%), scoring 4 points.

The most frequent adverse reaction of pegmolesatide was hypertension, with an incidence of 5.7%, scoring 2 points. The proportion of high-grade (≥ Grade 3) hepatotoxicity was 0.7%, electrolyte abnormality was 8.2%, and hypersensitivity reactions mainly presented as pruritus and conjunctivitis, with an incidence of 0.2%, scoring 2 points.

#### 3.2.2 Special populations

##### 3.2.2.1 Children

Clinical data on the use of long-acting ESAs in children with CKD are relatively limited. In particular, the safety and efficacy of long-acting ESAs in Chinese children with CKD remain unclear. Two RCTs from Europe and the United States found that darbepoetin alfa was safe and effective in correcting renal anemia in children with CKD aged 1–18 years, showing non-inferiority to short-acting ESAs, regardless of dialysis status (Bruce et al., 2022; Warady et al., 2006; Warady et al., 2018). Additionally, a prospective Phase IV observational study from Europe and the United States enrolled 319 children with CKD aged 0–16 years (4.1% under 1 year of age), further confirming the safety and efficacy of darbepoetin alfa in this population (Schaefer et al., 2016). Two multicenter, open-label Phase II clinical trials from Europe and the United States enrolled children with CKD aged 6–17 years and 3 months to 18 years, respectively, who were receiving other ESAs (Fischbach et al., 2018; Warady et al., 2023). The results showed that switching to CERA safely and effectively maintained Hb levels within the target range.

Darbepoetin alfa and CERA are applicable to pediatric patients, scoring 2 points each. Pegmolesatide has not been evaluated for clinical efficacy and safety in this population and lacks reliable literature, scoring 0 points.

##### 3.2.2.2 Elderly

No dose adjustment is required at treatment initiation in patients aged 65 years and older. RCTs of various long-acting ESAs conducted both domestically and internationally have included elderly patients aged 65 years and older, with this proportion exceeding 50% in some studies. No significant differences in safety or efficacy were observed between elderly and younger patients (Pfeffer et al., 2009; Liu et al., 2022; Chen et al., 2022). Darbepoetin alfa, CERA, and pegmolesatide are all suitable for use in elderly patients, with 1 point awarded to each.

##### 3.2.2.3 Pregnancy and lactation

No clinical studies have been conducted on the use of darbepoetin alfa, CERA, or pegmolesatide in pregnant or lactating women. Therefore, all three agents received a score of 0 points for this criterion.

##### 3.2.2.4 Hepatic insufficiency

Darbepoetin alfa is primarily metabolized and eliminated by the liver. Patients with concurrent active liver disease were excluded from all studies on darbepoetin alfa. Therefore, no data are available on its use in patients with hepatic impairment. Caution is recommended for CKD patients with hepatic impairment using darbepoetin alfa, scoring 0 points. A non-randomized, multicenter study indicated that severe hepatic impairment does not affect the pharmacokinetics of CERA. Thus, no dose adjustment is theoretically required in CKD patients with hepatic impairment treated with CERA (Kupcová et al., 2008), scoring 4 points. Regarding the use of pegmolesatide in patients with hepatic impairment, a population pharmacokinetic model analysis indicated that mild hepatic impairment does not affect the steady-state exposure to pegmolesatide (National Healthcare Security Administration, 2023-07), but its clinical significance remains unclear, and further studies are needed. This criterion was assigned 2 points.

##### 3.2.2.5 Renal insufficiency

Darbepoetin alfa, CERA, and pegmolesatide can be used in patients with renal impairment, with 4 points awarded to each agent.

#### 3.2.3 Drug interactions

Darbepoetin alfa may interact with substances that are highly bound to erythrocytes, such as cyclosporine and tacrolimus. Blood concentrations of these drugs should be monitored, and doses should be adjusted according to changes in hemoglobin when they are administered concomitantly. This criterion was assigned 2 points. No drug interactions have been identified with CERA in clinical studies, scoring 3 points. Similarly, no drug interactions have been reported for pegmolesatide in clinical studies, and this criterion was assigned 3 points.

#### 3.2.4 Others

This subcategory is worth a total of 1 point and is divided into three components weighted as follows: teratogenicity (0.4 points), reproductive toxicity (0.2 points), and carcinogenicity (0.4 points). Teratogenicity and carcinogenicity are assigned higher weights than general reproductive toxicity because they represent irreversible and severe adverse outcomes. For each component, a drug receives the full component score if no toxicity signal is identified in the available data and 0 points if a toxicity signal is present or if adequate data are unavailable (i.e., unevaluated).

Darbepoetin alfa was non-teratogenic but exhibited reproductive toxicity; carcinogenicity studies have not been performed, scoring 0.4 points.

CERA was non-teratogenic and non-reproductively toxic; however, carcinogenicity has not been evaluated, scoring 0.6 points.

Pegmolesatide was non-teratogenic but exhibited reproductive toxicity and carcinogenicity, scoring 0.4 points.

All three agents carry special prescribing warnings. ESAs may increase the risk of death, myocardial infarction, stroke, venous thromboembolism, vascular access thrombosis, and tumor progression or recurrence. Therefore, each received a score of 0 points for this criterion.

The safety score results are shown in Table 4.

**TABLE 4:** Safety score results.

Safety (27 points)		Grading criteria	Darbepoetin alfa	CERA	Pegmolesatide
Adverse reactions (9)	Moderate adverse reactions (4)				
	Incidence <0.1%	4	—	—	—
	Incidence ≥0.1% to <1%	3	—	—	—
	Incidence ≥1% to <10%	2	—	2	2
	Incidence ≥10%	1	1	—	—
	ADR occurrence data not available	0	—	—	—
	Severe adverse reactions (5)				
	Incidence <0.01%	5	—	—	—
	Incidence ≥0.01% to <0.1%	4	—	4	—
	Incidence ≥0.1% to <1%	3	—	—	—
	Incidence ≥1% to <10%	2	2	—	2
	Incidence ≥10%	1	—	—	—
	ADR occurrence data not available	0	—	—	—
Special populations (13 points)	Available for children (both 2; 1.5 for ages 1+; 1 for ages 6+; 0.5 for ages 12+	2	2	2	0
	The elderly (available 1; use with caution 0.5)	1	1	1	1
	Pregnant women (early pregnancy 1; during the first trimester 0.8; during the second trimester 0.5)	1	0	0	0
	Lactating women (available 1; use with caution 0.5)	1	0	0	0
	Hepatic dysfunction (severe available 4; moderate available 3; lightly available 2)	4	0	4	2
	Renal dysfunction (severe available 4; moderate available 3; lightly available 2)	4	4	4	4
Drug interaction (3 points)	No dosage adjustment is required	3	—	3	3
	Dosage adjustment required	2	2	—	—
	Prohibited to use at the same time	1	—	—	—
Others (2 points)	No teratogenicity, reproductive toxicity, or carcinogenicity	1	0.4	0.6	0.4
	No special medication warnings	1	0	0	0
Total safety score		—	12.4	20.6	14.4

### 3.3 Pharmaceutical properties (27 points)

#### 3.3.1 Pharmacological effects

Darbepoetin alfa is produced by modifying the molecular structure of epoetin alfa using recombinant DNA technology, thereby increasing the number of glycosylation sites to promote erythropoiesis. Darbepoetin alfa represents a “same mechanism, novel structure” incremental innovation: the advance lies in molecular engineering and long-acting pharmacokinetics rather than in a new target, pathway, or fundamentally altered receptor-binding properties. CERA is a chemically synthesized erythropoietin receptor activator that stimulates erythropoiesis by acting on erythropoietin receptors on bone marrow stem cells. Pegmolesatide is a branched PEGylated erythropoietin-mimetic peptide obtained by PEGylation of two linear peptides; it can activate erythropoietin receptors and stimulate EPO-dependent cell proliferation *in vitro*. Darbepoetin alfa scored 3 points for its well-established but non-novel mechanism of action, whereas CERA and pegmolesatide each scored 5 points for their innovative mechanisms of action.

#### 3.3.2 *In vivo* processes

The *in vivo* processes of the three long-acting ESAs are well characterized, with complete pharmacokinetic parameters, and each was awarded 5 points.

#### 3.3.3 Pharmacy and method of use

The main ingredients and excipients of the three ESAs are clearly identified, scoring 1 point each. Darbepoetin alfa and CERA are both fixed-dose prefilled syringes, which are not convenient for precise dose adjustment and rely on changing syringes of different specifications for dose titration, scoring 0.5 points. Pegmolesatide has specifications and packaging that meet clinical application and dose adjustment needs, scoring 1 point. All three ESAs can be administered subcutaneously, each scoring 0.5 points. During treatment, the dose should be adjusted according to changes in the patient’s Hb concentration, scoring 0.8 points. Darbepoetin alfa and CERA are administered once every 1–2 weeks, scoring 1.8 points, while pegmolesatide is administered subcutaneously once a month, scoring 2 points. None of the three drugs require administration at a specific time of day, each scoring 2 points. All should be used under the supervision and guidance of medical professionals with experience in the treatment of renal anemia, scoring 1 point each.

#### 3.3.4 Storage conditions

All three drugs require refrigerated storage at 2 °C–8 °C and protection from light, scoring 1 point each.

#### 3.3.5 Expiry date

Darbepoetin alfa has an expiry period of 24 months, scoring 1.5 points. CERA and pegmolesatide have an expiry period of 36 months, scoring 2 points each.

The results of the pharmaceutical property scores are presented in Table 5.

**TABLE 5:** Results of pharmacological properties scoring.

Pharmaceutical property (27 points)		Grading criteria	Darbepoetin alfa	CERA	Pegmolesatide
Pharmacological effects (5 points)	Precise mechanism of action and innovative mechanism of action or target point of action	5	—	5	5
	Precise mechanism of action	3	3	—	—
	Mechanism of action is unclear	1	—	—	—
Pharmacokinetics (5 points)	Well-defined *in vivo* process with complete pharmacokinetic parameters	5	5	5	5
	Well-defined *in vivo* process with incomplete pharmacokinetic parameters	3	—	—	—
	*In vivo* processes are unclear, or no pharmacokinetic studies are available	1	—	—	—
Pharmaceutical formulation and administration (multiple choice) (12 points)	Main ingredients and excipients (all specify 1; one specifies 0.5)	1	1	1	1
	Specification and packaging (all appropriate for clinical use/dose adjustment 1; one appropriate 0.5)	1	0.5	0.5	1
	Dosage forms (oral/inhalation/topical formulations 1; subcutaneous/intramuscular injections 0.5)	1	0.5	0.5	0.5
	The dose administered (fixed dose 2; dose to be adjusted during use 1.5; dose based on body mass or body surface area 1)	2	1.5	1.5	1.5
	Frequency of administration (once every 4 weeks or more 2; once every 1–4 weeks 1.8; once every 1 day to 1 week 1.5; twice a day 1; three or more times a day 0.5)	2	1.8	1.8	2
	Medication administration time (no need to administer at a specific time 2; need to administer at a specific time 1)	2	2	2	2
	Ease of use (self-administration without assistance 3; with help or training 2; administered by medical personnel 1)	3	1	1	1
Storage conditions (multiple choice) (3 points)	Storage at room temperature	2	—	—	—
	Storage in the shade	1.5	—	—	—
	Refrigerated/frozen storage	1	1	1	1
	No need for shade/light protection	1	—	—	—
Drug shelf life (2 points)	≥36 months	2	—	2	2
	≥24 months, <36 months	1.5	1.5	—	—
	≥12 person-months, <24 months	1	—	—	—
	<12 months	0.5	—	—	—
Pharmaceutical properties score		—	18.8	21.3	22

### 3.4 Economy (10 points)

The economic evaluation was conducted by comparing the differences in average daily treatment costs between the three ESAs and the main indication drugs with the same generic name and alternative drugs. The unit prices of drugs were obtained from the latest prices available online on local procurement platforms, with information as of 31 March 2026. However, drug costs are context-specific and may vary by region, procurement channel, reimbursement level, and time. Accordingly, the economic estimates should be interpreted as indicative rather than definitive, and institutions in different countries and regions are encouraged to recalculate them using local price data. For drugs with the same generic name, all three agents are originator drugs, with no other generic equivalents available; therefore, each received a score of 3 for this criterion. Alternative drugs for the main indication: the drug with the lowest average daily treatment cost scores 7 points, and the score of the evaluated drug = (lowest average daily treatment cost/average daily treatment cost of the evaluated drug) × 7. Assuming a patient weight of 70 kg, all three ESAs are available as prefilled syringes—single-use, ready-to-administer dosage forms. Darbepoetin alfa had the lowest average daily treatment cost, scoring 7 points; CERA scored 5.99 points; and pegmolesatide scored 4.14 points.

Detailed scores are shown in Tables 6, 7.

**TABLE 6:** Basic economy information.

Generic name	Unit price (¥) (dosage forms and strengths)	Therapeutic dose	Frequency of administration	Average daily treatment cost (¥)
Darbepoetin alfa	115.26 (40 μg/0.5 mL)	0.45 μg/kg	q.w	16.47
CERA	269.41 (50 μg/0.3 mL)	0.6 μg/kg	q.2w	19.24
Pegmolesatide	779 (4.0 mg/1 mL)	0.04 mg/kg	q.4w	27.82

**TABLE 7:** Economy score results.

Economy (10 points)		Grading criteria	Darbepoetin alfa	CERA	Pegmolesatide
Same generic name drug (3 points)	Daily treatment cost of the lowest-priced drug is 3 points; evaluation drug score = lowest daily treatment cost/average daily treatment cost of the evaluated drug × 3	3	3	3	3
Alternative drugs with the main indications (7 points)	Daily treatment cost of the lowest-priced drug is 7 points; Evaluation drug score = lowest daily treatment cost/average daily treatment cost of the evaluated drug × 7	7	7	5.99	4.14
Total score for economics		—	10	8.99	7.14

### 3.5 Other attributes (8 points)

#### 3.5.1 National health insurance and national essential drug characteristics

All three drugs are included in the negotiated drugs section of the National Health Insurance catalog and categorized as Category B, which entails payment limitations, with each scoring 1.5 points. None of them are included in the National Essential Medicines List, scoring 0 points.

#### 3.5.2 National centralized drug procurement and original research drugs

None of the three agents are included in the National Centralized Procurement Program, each scoring 0 points. All three agents are original drugs, each scoring 1 point.

#### 3.5.3 Manufacturer status

The rationale for including this indicator is to leverage publicly available industry rankings—specifically, the Top 50 Global Pharmaceutical Companies ranking published by Pharmaceutical Manager and the Top 100 Pharmaceutical Industry Companies ranking released by the Ministry of Industry and Information Technology (MIIT)—as an indirect proxy for assessing a manufacturer’s overall production quality-control system, production capacity reserves, and supply-chain stability. This directly addresses the practical operational need of healthcare institutions for a sustained and reliable drug supply. It should be emphasized that enterprise sales scale is in no way equated with the clinical value or product quality of the individual agent under evaluation.

The “manufacturer status” item (weight: 1 point) falls under the “Other Attributes” dimension and is not a core metric for judging a drug’s clinical efficacy, safety, or quality. The core evaluation of each drug is conducted across three principal dimensions: efficacy, safety, and pharmaceutical properties. This particular criterion accounts for only 1 of the 100 total points (1%), rendering it a secondary factor within the overall assessment that does not drive the final ranking.

Darbepoetin alfa is manufactured by Terumo Corporation, Kofu Factory, which is not among the world’s top 50 pharmaceutical companies by sales volume, scoring 0 points. CERA is manufactured by Roche Pharma (Schweiz) AG, which ranked fourth among the world’s top 50 pharmaceutical companies by sales volume in 2025, scoring 1 point. Pegmolesatide is manufactured by Jiangsu Hansoh Pharmaceutical Group Co., Ltd., which ranked 30th on the 2024 Top 100 Pharmaceutical Enterprises List issued by the Ministry of Industry and Information Technology, receiving a score of 1 point.

#### 3.5.4 Global utilization

Darbepoetin alfa and CERA are marketed in China, the United States, Europe, and Japan, scoring 1 point. Pegmolesatide is currently only sold in China, scoring 0 points.

The other attributes’ score results are shown in Table 8.

**TABLE 8:** Other attributes’ score results.

Other attributes (8 points)		Grading criteria	Darbepoetin alfa	CERA	Pegmolesatide
National Health Insurance (3 points)	National medical insurance category A, no payment limitations	3	—	—	—
	National medical insurance category A with payment limitations	2.5	—	—	—
	National medical insurance category B, no payment limitations	2	—	—	—
	National medical insurance category B with payment limitations	1.5	1.5	1.5	1.5
	Drugs included in the commercial health insurance innovative medicine list	1	—	—	—
National essential drugs (1 points)	National essential drugs without Δ requirement	1	0	0	0
	National essential drugs with Δ requirements	0.5	—	—	—
National Centralized Drug Procurement (1 point)	Selected drugs for centralized national procurement	1	0	0	0
Originator/reference/consistency evaluation (1 point)	Drug of origin/reference drug	1	1	1	1
	Generic drugs through consistency evaluation	0.5	—	—	—
Manufacturer ranking (1 point)	The world’s top 50 pharmaceutical manufacturers in terms of sales volume/Top 100 Pharmaceutical Industry published by Ministry of Industry and Information Technology	1	0	1	1
Global usage (1 point)	Available in China, the United States, Europe, and Japan	1	1	1	0
	Domestic and international sales	0.5	—	—	—
Total score for other attributes		—	3.5	4.5	3.5

The “Δ” sign indicates that the drug should be used by a physician with corresponding prescription qualifications or under the guidance of a specialist physician and that monitoring and evaluation of its use should be strengthened.

## 4 Discussion

As first-line treatments for renal anemia, long-acting ESAs exhibit notable differences in clinical efficacy and drug cost, while the evidence remains relatively limited. According to the Rapid Guidelines for Drug Evaluation and Selection in Chinese Medical Institutions (Third Edition), a comprehensive evaluation was performed on the core attributes of the three long-acting ESAs, including pharmaceutical properties, effectiveness, safety, and economics, as well as policy attributes related to national drug support. We assessed the clinical value and application potential of the drugs to provide a reference for clinical drug selection in hospitals and for patients.

As presented in Table 9, among the three long-acting ESAs evaluated, CERA achieved the highest total score, followed by darbepoetin alfa and pegmolesatide. All three agents scored above 70 points and are therefore strongly recommended for use. It should be emphasized that these rankings reflect a synthesis of evidence within a predefined scoring framework and should not be interpreted as direct comparative effectiveness evidence or as establishing that one drug is inherently clinically superior to another (Zhao et al., 2026).

**TABLE 9:** Final total score results.

Evaluation dimension	Darbepoetin alfa	CERA	Pegmolesatide
Efficacy	27	25	26
Safety	12.4	20.6	14.4
Pharmaceutical property	18.8	21.3	22
Economy	10	8.99	7.14
Other attributes	3.5	4.5	3.5
Total score	71.7	80.39	73.04

In the efficacy dimension, the three long-acting ESAs received similar scores. The evaluation of clinical efficacy was based on predefined scoring criteria, according to which the primary and secondary endpoints were assessed for superiority or non-inferiority based on drug labels and clinical study results. Among the three agents, darbepoetin alfa exhibited robust clinical efficacy and achieved the highest score, reflecting its prominent therapeutic advantages. Multiple multicenter randomized clinical trials conducted in Asia, Europe, and North America have also demonstrated the efficacy and safety of darbepoetin alfa in the treatment of renal anemia among patients undergoing hemodialysis and peritoneal dialysis (Vanrenterghem et al., 2002; Sinha et al., 2019; Fang and Chang, 2009), with findings generally consistent with guideline recommendations (DelVecchio et al., 2026). CERA demonstrated non-inferiority in the primary efficacy endpoint, indicating a certain efficacy profile; however, differences between agents in the primary or secondary endpoints influenced the final evaluation conclusions. Although pegmolesatide showed favorable clinical efficacy, its score was relatively lower, primarily due to its recent market entry as a Chinese innovative drug and the lack of support from international guidelines.

In the safety dimension, CERA does not require initial dose adjustment or modification of dose-titration regimens in patients with any degree of hepatic impairment and has a relatively low incidence of moderate and severe adverse reactions, thus achieving the highest score. A targeted sensitivity analysis was performed by varying the weight of the hepatic impairment sub-criterion by ±50%. CERA remained consistently ranked first across all scenarios (scores 78.39–82.39), followed by pegmolesatide (72.04–74.04) and darbepoetin alfa (71.70), with no rank reversal observed. All three agents remained above the 70-point strongly recommended threshold, confirming that the overall ranking is robust to changes in this weighting. For use in special populations, the evaluation was conducted in combination with expert consensus to more accurately reflect the clinical application of the three long-acting ESAs. Pegmolesatide received a slightly lower score due to the lack of safety data in pediatric patients. Several case reports and clinical studies have indicated that pure red cell aplasia occurring after treatment with short-acting ESAs remains responsive to long-acting ESA therapy (Macdougall et al., 2009; Lim et al., 2013), although further in-depth investigation is warranted.

In terms of pharmaceutical characteristics, pegmolesatide was administered once monthly, improving patient adherence and contributing to a relatively high score. The three agents had identical storage conditions. CERA and pegmolesatide had advantages in terms of shelf life, which may indicate better suitability for long-term use and storage, thus resulting in relatively high scores.

In the economic dimension, all three agents are available as prefilled syringes with single-use, individually packaged doses. Darbepoetin alfa demonstrated the most favorable price advantage, which may reduce the economic burden on patients while ensuring drug safety and efficacy. Results from a randomized controlled trial showed that, over a 12-month observation period, the median cost was 4,179 USD for EPO and 2,303 USD for darbepoetin alfa, with a cost difference of 1,876 USD (*p =* 0.02). The annual per-patient cost of darbepoetin alfa was 1,876 USD lower than that of EPO (Woodland et al., 2017). Another brief report from a hemodialysis clinic in Japan indicated that darbepoetin alfa was more cost-effectiveness for the treatment of renal anemia than EPO (Nakagawa, 2008). These studies suggest that darbepoetin alfa is more cost-effective than EPO for renal anemia in hemodialysis patients, which is consistent with the results of the present study. After darbepoetin alfa was included in China’s National Reimbursement Drug List, its price decreased to approximately one-third of the original level, which may be the main reason for the better economic performance of darbepoetin alfa in our study. The economic dimension of this evaluation primarily assessed drug acquisition costs, consistent with the rapid guideline methodology. However, several indirect economic factors merit discussion. First, the three agents differ substantially in dosing frequency: darbepoetin alfa once weekly, CERA once every 2 weeks, and pegmolesatide once every 4 weeks. The extended dosing intervals of CERA and pegmolesatide may reduce outpatient visits and nursing workload; however, all three agents are available in prefilled syringe formulations, which partially offsets this advantage. Second, all three agents can reduce transfusion requirements, thereby not only lowering direct costs but also reducing transfusion-related risks such as alloimmunization and infection. Third, monitoring costs are similar across the three agents as all require regular hemoglobin monitoring. Future studies should incorporate these indirect costs and savings to enable a comprehensive pharmacoeconomic analysis.

In the other attributes dimension, darbepoetin alfa and pegmolesatide received relatively lower scores, attributed to the fact that the manufacturer of darbepoetin alfa is not among the world’s top 50 pharmaceutical companies by sales volume, and pegmolesatide is currently marketed only in China. CERA achieved the highest score in this dimension, reflecting the global standing of its manufacturer, Roche (ranked fourth worldwide), as well as its market availability in China, the United States, Europe, and Japan.

With the successive clinical introduction of long-acting ESAs—including darbepoetin alfa, CERA, and pegmolesatide—structural modifications have extended their *in vivo* half-lives, reducing dosing frequency to once weekly or even once monthly. By substantially lowering the treatment burden while maintaining efficacy and safety, these agents improve patient adherence and are advancing the management of renal anemia into a new era of precision, efficiency, and safety (Kidney Disease: Improving Global Outcomes KDIGO Anemia Work Group, 2026). We conducted a comprehensive evaluation of three long-acting ESAs, including commonly used darbepoetin alfa and CERA, as well as the newly marketed pegmolesatide. The aim was to clarify whether traditional drugs still maintain clinical advantages and perform a comprehensive comparison with novel agents. The results demonstrated that darbepoetin alfa and CERA exhibited outstanding performance in terms of economics and safety. In the current economic context of China, darbepoetin alfa also demonstrates a cost-effectiveness advantage over roxadustat for the treatment of anemia in patients with CKD (Xu et al., 2024). Locatelli et al. (2019) followed 2,818 patients with CKD for a median of 3.4 years and found that, compared with short-acting ESAs, CERA did not increase the risk of adverse cardiovascular events or all-cause mortality in a large CKD population predominantly comprising dialysis patients—a finding consistent with the conclusions of prior meta-analyses (Chung et al., 2023). With a longer marketing history and extensive clinical application, these agents have established a robust evidence base for efficacy and safety, supported by numerous clinical trials and real-world research data (Bernieh et al., 2014). They show definite efficacy in improving renal anemia and impose a lower economic burden on patients under the current medical insurance policy.

The novel agent also demonstrates significant advantages. Pegmolesatide is the world’s first highly selective erythropoietin receptor peptide agonist and the only long-acting monthly ESA approved in China. Modified with polyethylene glycol, this agent has a significantly prolonged half-life, enabling once-every-4-week dosing that achieves stable hemoglobin target levels (Xie et al., 2024). Such a long dosing interval effectively improves patient adherence to treatment. However, the new agent still faces certain challenges, including a relatively short time on the market, limited clinical data, and higher direct treatment costs for patients. As clinical evidence continues to accumulate, this novel agent is expected to exhibit further potential advantages. It should be acknowledged that HIF-PHIs are now recommended alongside ESAs in several renal anemia guidelines but were not included in this category-specific rapid HTA, which was predefined by the hospital pharmacy and therapeutics committee to focus on long-acting ESAs. Unlike ESAs with a well-established safety profile, HIF-PHIs, despite the advantage of oral administration, remain limited by concerns over thrombotic events and tumor progression, and their long-term safety data are still maturing. The present ranking therefore reflects only the relative comparison among the three long-acting ESAs and should not be interpreted as excluding HIF-PHIs. Future studies should incorporate this emerging class to provide a more comprehensive formulary evaluation. In conclusion, the selection of clinical medication should be based on the patient’s condition, treatment objectives, and medical resource status, with comprehensive consideration of efficacy, safety, and economy.

This evaluation framework, while conceived within the Chinese healthcare context, is designed with transferability as a core principle, offering a structured tool for drug evaluation and selection in medical institutions worldwide. The universally applicable core dimensions—pharmaceutical properties, efficacy, and safety—retain direct relevance across most healthcare settings, with their assessment criteria and baseline weights readily adaptable to maintain clinical and scientific rigor. For context-specific indicators that reflect distinctly Chinese policy environments—such as the National Essential Medicines List, the National Medical Insurance catalog, and centralized drug procurement—evaluators in other regions should substitute locally relevant equivalents (e.g., national formulary status, local procurement policies, or regional reimbursement frameworks) to align the model with their respective health policies and healthcare structures. Accordingly, this study not only provides a scientific basis for drug selection and decision-making within Chinese medical institutions but also offers a transferable evaluation methodology for healthcare decision-making in other regions.

## 5 Limitations

Although this evaluation provides a relatively comprehensive decision-making basis for drug selection and rational clinical use in medical institutions, several limitations should be acknowledged: 1. a degree of subjectivity may exist in the evaluation process. Weights were determined through a modified Delphi consensus process, with certain scoring outcomes incorporating subjective judgment. The primary and secondary efficacy endpoints used to assess drug efficacy vary across studies, and no uniform standards have been established in the relevant literature. 2. The drug scoring results are dynamic. As evidence-based data are updated, drug policies are adjusted, or package inserts are revised, the evaluation findings should be refined and updated accordingly. A complete cost-effectiveness analysis incorporating indirect costs would provide a more comprehensive economic evaluation and should be considered in future research. 3. In comparative studies of clinical efficacy and adverse reactions, there are still certain evidence gaps for the three long-acting ESAs. Most existing studies involve indirect comparisons and lack direct head-to-head comparative trials. Future research with rigorous design, larger sample sizes, and longer follow-up periods is needed to address these gaps.

## 6 Conclusion

Based on this multi-dimensional evaluation framework, all three long-acting ESAs scored above the strong recommendation threshold (>70 points) defined in the Rapid Guideline, supporting their consideration for inclusion in the formulary within the Chinese healthcare context. CERA achieved the highest total score within this framework, while darbepoetin alfa and pegmolesatide demonstrated comparable overall performance, each with differentiated advantages in economy and pharmaceutical characteristics, respectively. These findings should be interpreted as a structured synthesis of evidence to inform prescribing decisions under specific institutional, economic, and patient-population assumptions, rather than as establishing a universally superior therapeutic regimen. Individual treatment selection should be guided by patient characteristics, iron status, dosing considerations, and local resource availability. This health technology assessment provides evidence-based guidance for the selection and rational use of long-acting ESAs and may also serve as a reference for hospital drug selection processes in other countries.

## Data Availability

The original contributions presented in the study are included in the article/Supplementary Material; further inquiries can be directed to the corresponding author.

## References

[B1] Babitt J. L. Lin H. Y. (2012). Mechanisms of anemia in CKD. J. Am. Soc. Nephrol. 23 (10), 1631–1634. 10.1681/ASN.2011111078 22935483 PMC3458456

[B2] Bello A. K. Okpechi I. G. Levin A. Ye F. Damster S. Arruebo S. (2024). An update on the global disparities in kidney disease burden and care across world countries and regions. Lancet Glob. Health 12 (3), e382–e395. 10.1016/S2214-109X(23)00570-3 38365413

[B3] Bernieh B. Abouchacra S. Boobes Y. Al Hakim M. R. Nagelkerke N. Chaaban A. (2014). Comparison between short- and long-acting erythropoiesis-stimulating agents in hemodialysis patients: target hemoglobin, variability, and outcome. Int. Urol. Nephrol. 46 (2), 453–459. 10.1007/s11255-013-0640-7 24448756 PMC3932161

[B4] Bhandari S. Spencer S. Oliveira B. Mikhail A. Brooks O. Bryant G. (2025). UK kidney association clinical practice guideline: update of anaemia of chronic kidney disease. BMC Nephrol. 26 (1), 193. 10.1186/s12882-025-04115-1 40240983 PMC12004666

[B5] Bruce G. Schulga P. Reynolds B. C. (2022). Use of erythropoiesis-stimulating agents in children with chronic kidney disease: a systematic review. Clin. Kidney J. 15 (8), 1483–1505. 10.1093/ckj/sfac058 35892014 PMC9308099

[B6] Chen N. Xing C. Niu J. Liu B. Fu J. Zhao J. (2022). Darbepoetin alfa versusepoetin alfa injection for treating anemia of Chinese hemodialysis patients with chronic kidney failure: a randomized, open-label, parallel-group, non-inferiority phase III trail. Chronic Dis. Transl. Med. 8 (1), 59–70. 10.1002/cdt3.13 35620165 PMC9128564

[B7] Chung E. Y. Palmer S. C. Saglimbene V. M. Craig J. C. Tonelli M. Strippoli G. F. (2023). Erythropoiesis-stimulating agents for anaemia in adults with chronic kidney disease: a network meta-analysis. Cochrane Database Syst. Rev. 2 (2), CD010590. 10.1002/14651858.CD010590.pub3 36791280 PMC9924302

[B8] Clinical practice guidelines for management of chronic kidney disease during peridialysis in China (2025) Working Group of the Kidney Disease Dialysis Special Committee of the China Association of Non-Public Medical Institutions (2025). Clinical practice guidelines for the management of peri-dialysis chronic kidney disease in China (2025 edition). Chin. J. Nephrol. 41 (10), 788–810. 10.3760/cma.j.cn441217-20250506-00504

[B9] Cody J. D. Hodson E. M. (2016). Recombinant human erythropoietin *versus* placebo or no treatment for the anaemia of chronic kidney disease in people not requiring dialysis. Cochrane Database Syst. Rev. 2016 (1), CD003266. 10.1002/14651858.CD003266.pub3 26790135 PMC7144724

[B10] Consensus Expert Group, Dialysis Professional Committee of the Chinese Non-Public Hospital Association (2024). Chinese expert consensus on long-acting erythropoiesis-stimulating agents for the treatment of renal anemia (2024 edition). Chin. J. Nephrol. 40 (2), 146–157. 10.3760/cma.j.cn441217-20231025-01037

[B11] DelVecchio L. Cases A. Eisenga M. F. Małyszko J. Barratt J. Bolignano D. (2026). KDIGO 2026 clinical practice guideline for anemia in chronic kidney disease (CKD): a commentary from the european renal best practice (ERBP). Nephrol. Dial. Transpl. 41, 1554–1572. 10.1093/ndt/gfag014 PMC13423824 41604211

[B12] Eriksson D. Goldsmith D. Jackson J. van Nooten F. Teitsson S. (2016). Cross-sectional survey in CKD patients across Europe describing the association between quality of life and anaemia. BMC Nephrol. 17 (1), 97. 10.1186/s12882-016-0312-9 27460779 PMC4962379

[B13] Expert Group of Clinical Management Guidelines for Delaying the Progression of Chronic Kidney Disease (2025 Edition). (2025). Clinical management guidelines for delaying the progression of chronic kidney disease (2025 edition). Chin. J. Nephrol. 41 (6), 455–488. 10.3760/cma.j.cn441217-20250121-00143

[B14] Fang Y. W. Chang C. H. (2009). Subcutaneous administration of darbepoetin alfa effectively maintains hemoglobin concentrations at extended dose intervals in peritoneal dialysis patients. Perit. Dial. Int. 29 (2), 199–203. 10.1177/089686080902900215 19293358

[B15] Fischbach M. Wühl E. Reigner S. Morgan Z. Schaefer F. (2018). Efficacy and long-term safety of C.E.R.A. maintenance in pediatric hemodialysis patients with anemia of CKD. Clin. J. Am. Soc. Nephrol. 13 (1), 81–90. 10.2215/CJN.03570417 29097481 PMC5753305

[B16] Hörl W. H. (2013). Differentiating factors between erythropoiesis-stimulating agents: an update to selection for anaemia of chronic kidney disease. Drugs 73 (2), 117–130. 10.1007/s40265-012-0002-2 23338536

[B17] Hoshino J. Muenz D. Zee J. Sukul N. Speyer E. Guedes M. (2020). CKDopps investigators. Associations of hemoglobin levels with health-related quality of life, physical activity, and clinical outcomes in persons with stage 3-5 nondialysis CKD. J. Ren. Nutr. 30 (5), 404–414. 10.1053/j.jrn.2019.11.003 31980326

[B18] Kidney Disease: Improving Global Outcomes (KDIGO) Anemia Work Group (2026). KDIGO 2026 clinical practice guideline for the management of anemia in chronic kidney disease (CKD). Kidney Int. 109 (1S), S1–S99. 10.1016/j.kint.2025.06.006 41485812

[B19] Kupcová V. Sperl J. Pannier A. Jordan P. Dougherty F. C. Reigner B. (2008). The effect of severe hepatic impairment on the pharmacokinetics and haematological response of C.E.R.A. Curr. Med. Res. Opin. 24 (7), 1943–1950. 10.1185/03007990802176467 18513461

[B20] Li M. Huang S. Li J. Hu X. Chen J. (2025). Health technology assessment: evaluation of 8 CGRP-targeted therapy drugs for the treatment of migraine. Drug Des. Devel. Ther. 19, 1231–1247. 10.2147/DDDT.S499848 39991088 PMC11847418

[B21] Lim S. K. Bee P. C. Keng T. C. Chong Y. B. (2013). Resolution of epoetin-induced pure red cell aplasia 2 years later, successful re-challenge with continuous erythropoiesis receptor stimulator. Clin. Nephrol. 80 (3), 227–230. 10.5414/CN107456 23298519

[B22] Liu B. Chen N. Zhao J. Yin A. Wu X. Xing C. (2022). Efficacy and safety of darbepoetin alfa replacing epoetin alfa injection for the treatment of renal anemia in Chinese hemodialysis patients: a randomized, open-label, parallel-group, noninferiority phase III trial. Chronic Dis. Transl. Med. 8 (2), 134–144. 10.1002/cdt3.23 35774427 PMC9215713

[B23] Locatelli F. Choukroun G. Truman M. Wiggenhauser A. Fliser D. (2016). Once-monthly continuous erythropoietin receptor activator (C.E.R.A.) in patients with hemodialysis-dependent chronic kidney disease: pooled data from phase III trials. Adv. Ther. 33 (4), 610–625. 10.1007/s12325-016-0309-6 26965694 PMC4846713

[B24] Locatelli F. Hannedouche T. Fishbane S. Morgan Z. Oguey D. White W. B. (2019). Cardiovascular safety and all-cause mortality of methoxy polyethylene glycol-epoetin beta and other erythropoiesis-stimulating agents in anemia of CKD: a randomized noninferiority trial. Clin. J. Am. Soc. Nephrol. 14 (12), 1701–1710. 10.2215/CJN.01380219 31420350 PMC6895480

[B25] Lv J. Fu H. Zhou Y. Li W. Zhang J. Zhou Z. (2025). Rapid health technology assessment of six non-ergot dopamine-receptor agonists for the treatment of early Parkinson's disease. Front. Pharmacol. 16, 1648833. 10.3389/fphar.2025.1648833 41480386 PMC12753440

[B26] Macdougall I. C. Rossert J. Casadevall N. Stead R. B. Duliege A. M. Froissart M. (2009). A peptide-based erythropoietin-receptor agonist for pure red-cell aplasia. N. Engl. J. Med. 361 (19), 1848–1855. 10.1056/NEJMoa074037 19890127

[B27] Nakagawa T. (2008). Darbepoetin alpha is highly cost-effective compared with epoetin alpha in the treatment of renal anemia: a brief report from a hemodialysis clinic in Japan. Ther. Apher. Dial. 12 (6), 531–532. 10.1111/j.1744-9987.2008.00644.x 19140854

[B28] National Healthcare Security Administration (2023). Package insert of pegmolesatide injection [EB/OL]. Available online at: http://www.nhsa.gov.cn/attach/Ypsn2023/YPSW202300263/YPSW202300263-W1(ppt).pdf (Accessed March 31, 2026).

[B29] NCI (2011). Common terminology criteria for adverse events (CTCAE). Version 6. Bethesda, MD: US Department of Health and Human Services, National Institutes of Health, National Cancer Institute.

[B30] Pfeffer M. A. Burdmann E. A. Chen C. Y. Cooper M. E. de Zeeuw D. Eckardt K. U. (2009). A trial of darbepoetin alfa in type 2 diabetes and chronic kidney disease. N. Engl. J. Med. 361 (21), 2019–2032. 10.1056/NEJMoa0907845 19880844

[B31] Qian J. Chen N. Mei C. Liu W. Hou F. Shi W. (2012). Efficacy, safety and tolerability of continuous erythropoietin receptor activator administered intravenously for correcting anemia in dialysis patients with chronic kidney disease. Chin. J. Nephrol. 28 (11), 847–852. 10.3760/cma.j.issn.1001-7097.2012.11.006

[B32] Renal Anemia Guidelines Working Group, Chinese Society of Nephrology Physicians, Chinese Medical Doctor Association (2021). Clinical practice guidelines for the diagnosis and treatment of renal anemia in China. Natl. Med. J. China 101 (20), 1463–1502. 10.3760/cma.j.cn112137-20210201-00309

[B33] Saglimbene V. M. Palmer S. C. Ruospo M. Natale P. Craig J. C. Strippoli G. F. (2017). Continuous erythropoiesis receptor activator (CERA) for the anaemia of chronic kidney disease. Cochrane Database Syst. Rev. 8 (8), CD009904. 10.1002/14651858.CD009904.pub2 28782299 PMC6483666

[B34] Schaefer F. Hoppe B. Jungraithmayr T. Klaus G. Pape L. Farouk M. (2016). Safety and usage of darbepoetin alfa in children with chronic kidney disease: prospective registry study. Pediatr. Nephrol. 31 (3), 443–453. 10.1007/s00467-015-3225-0 26482252 PMC4756039

[B35] Sinha S. D. Bandi V. K. Bheemareddy B. R. Thakur P. Chary S. Mehta K. (2019). Efficacy, tolerability and safety of darbepoetin alfa injection for the treatment of anemia associated with chronic kidney disease (CKD) undergoing dialysis: a randomized, phase-III trial. BMC Nephrol. 20 (1), 90. 10.1186/s12882-019-1209-1 30866856 PMC6417108

[B36] Toft G. Heide-Jørgensen U. van Haalen H. James G. Hedman K. Birn H. (2020). Anemia and clinical outcomes in patients with non-dialysis dependent or dialysis dependent severe chronic kidney disease: a Danish population-based study. J. Nephrol. 33 (1), 147–156. 10.1007/s40620-019-00652-9 31587136 PMC7007417

[B37] Vanrenterghem Y. Bárány P. Mann J. F. Kerr P. G. Wilson J. Baker N. F. (2002). Randomized trial of darbepoetin alfa for treatment of renal anemia at a reduced dose frequency compared with rHuEPO in dialysis patients. Kidney Int. 62 (6), 2167–2175. 10.1046/j.1523-1755.2002.00657.x 12427142

[B38] Wang L. Xu X. Zhang M. Hu C. Zhang X. Li C. (2023). Prevalence of chronic kidney disease in China: results from the sixth China chronic disease and risk factor surveillance. JAMA Intern. Med. 183 (4), 298–310. 10.1001/jamainternmed.2022.6817 PMC9941971 36804760

[B39] Warady B. A. Arar M. Y. Lerner G. Nakanishi A. M. Stehman-Breen C. (2006). Darbepoetin alfa for the treatment of anemia in pediatric patients with chronic kidney disease. Pediatr. Nephrol. 21 (8), 1144–1152. 10.1007/s00467-006-0071-0 16724235

[B40] Warady B. A. Barcia J. Benador N. Jankauskiene A. Olson K. Podracka L. (2018). *De novo* weekly and biweekly darbepoetin alfa dosing in pediatric patients with chronic kidney disease. Pediatr. Nephrol. 33 (1), 125–137. 10.1007/s00467-017-3758-5 28815341

[B41] Warady B. A. MeyerReigner S. Tirodkar C. Drozdz D. (2023). Subcutaneous C.E.R.A. for the maintenance treatment of anemia in pediatric patients with CKD: a phase 2, open-label, single-arm, multicenter study. Am. J. Kidney Dis. 81 (6), 684–694.e1. 10.1053/j.ajkd.2022.11.006 36587890

[B42] Woodland A. L. Murphy S. W. Curtis B. M. Barrett B. J. (2017). Costs associated with intravenous Darbepoetin *versus* epoetin therapy in hemodialysis patients: a randomized controlled trial. Can. J. Kidney Health Dis. 30 (4), 2054358117716461. 10.1177/2054358117716461 28717516 PMC5502937

[B43] Xie J. Yang A. Qiu H. Peng X. Lu W. Huang X. (2024). Randomized trial of pegmolesatide for the treatment of anemia in patients with nondialysis CKD. Kidney Int. Rep. 10 (3), 720–729. 10.1016/j.ekir.2024.12.002 PMC11993201 40225393

[B44] Xu W. Guo M. Liu R. Cui X. (2024). Cost-effectiveness analysis of roxadustat and darbepoetin alfa for anemia treatment in dialysis-dependent chronic kidney disease patients. Her. Med. 43 (12), 2021–2025. 10.3870/j.issn.1004-0781.2024.12.024

[B45] Zhang P. Jiang Y. Xu C. Zhou L. Zheng H. Xie D. (2023). Pegmolesastide for the treatment of anemia in patients undergoing dialysis: a randomized clinical trial. EClinical Med. 65, 102273. 10.1016/j.eclinm.2023.102273 PMC10632410 37954906

[B46] Zhao Z. Dong Z. Liu J. (2023). A quick guideline for drug evaluation and selection in Chinese medical institutions (the second edition). Her. Med. 4, 447–456. 10.3870/j.issn.1004-0781.2023.04.001

[B47] Zhao Z. Dong Z. Liu J. (2026). A quick guideline for drug evaluation and selection in Chinese medical institutions (the third ddition). Her. Med. 45 (01), 1–9. 10.3870/j.issn.1004-0781.2026.01.001

