# Supplementary material for: Health technology assessment of long-acting erythropoiesis-stimulating agents for the treatment of renal anemia in China
Source: Front Pharmacol. 2026 Oct 2;17:1878826. doi: 10.3389/fphar.2026.1878826 (PMC13672644; doi:10.3389/fphar.2026.1878826)
Supplement: Supplementary file 1 [file Table1.docx]

**Supplementary Table 1 Evaluation dimensions, weights, and standardized scoring criteria (Zhao et al., 2026)**

| **Evaluation dimensions and corresponding weights （100 points）** | | | **Scoring criteria** |
| --- | --- | --- | --- |
| **Efficacy (28 points)** | Indications (8 points) | Disease Severity（3）： |  |
|  |  | Very high risk. The disease may be life‑threatening at any time. | 3 |
|  |  | High risk. Disease progression may be life‑threatening or cause permanent impairment. | 2 |
|  |  | Low risk. The disease is self‑limiting or progresses slowly, with little impact on the patient’s life expectancy. | 1 |
|  |  | VEN（5）： |  |
|  |  | Clinically necessary, vital drugs | 5 |
|  |  | Clinical need, essential drugs | 3 |
|  |  | More medicines available, nonessential drugs | 1 |
|  | Guide recommendation (10 points) | Strong recommendation: Diagnostic and treatment criteria, consensus statements / administrative measures issued by national health administrative departments, guidelines with Level I recommendation(A-level evidence: 10, B-level evidence: 9, C-level evidence: 8, others: 7) | 10 |
|  |  | Weak recommendation(A-level evidence: 6, B-level evidence: 5, C-level evidence and others: 4) | 6 |
|  |  | Expert consensus recommendation(Consensus issued by national-level academic societies) | 3 |
|  | Clinical efficacy (10 points) | The primary efficacy endpoint indicators | 5 |
|  |  | The secondary efficacy endpoint indicators | 3 |
|  |  | Can provide clinical efficacy data | 2 |
| **Safety (27 points)** | Adverse reactions (9) | Moderate adverse reactions（4）: |  |
|  |  | Incidence <0.1% | 4 |
|  |  | Incidence ≥0.1% ~ <1% | 3 |
|  |  | Incidence ≥1% ~ <10% | 2 |
|  |  | Incidence ≥10% | 1 |
|  |  | ADR occurrence data not available | 0 |
|  |  | Severe adverse reactions（5）: |  |
|  |  | Incidence < 0.01% | 5 |
|  |  | Incidence ≥0.01%~<0.1% | 4 |
|  |  | Incidence ≥0.1%~<1% | 3 |
|  |  | Incidence ≥1% to <10% | 2 |
|  |  | Incidence ≥10% | 1 |
|  |  | ADR occurrence data not available | 0 |
|  | Special populations  (13 points) | Available for children (both 2; 1.5 for ages 1+; 1 for ages 6+; 0.5 for ages 12+. | 2 |
|  |  | The elderly (available 1; use with caution 0.5) | 1 |
|  |  | Pregnant women (early pregnancy 1; during the first trimester 0.8; during the second trimester 0.5). | 1 |
|  |  | Lactating women (available 1; use with caution 0.5) | 1 |
|  |  | Hepatic dysfunction (severe available 4, moderate  available 3. Lightly available 2) | 4 |
|  |  | Renal dysfunction (severe available 4, moderate  available 3. Lightly available 2) | 4 |
|  | Drug interaction (3 points) | No dosage adjustment is required | 3 |
|  |  | Dosage adjustment required | 2 |
|  |  | Prohibited to use at the same time | 1 |
|  | Other (2 points) | No teratogenicity 、reproductive toxicity、or carcinogenicity | 1 |
|  |  | No special medication warnings | 1 |
| **Pharmaceutical properties (27 points)** | Pharmacological  effects (5 points) | Precise mechanism of action, and innovative mechanism of action or target point of action | 5 |
|  |  | Precise mechanism of action | 3 |
|  |  | Mechanism of action are unclear | 1 |
|  | Pharmacokinetics (5 points) | Well-defined in vivo process with complete pharmacokinetic parameters | 5 |
|  |  | Well-defined in vivo process with incomplete pharmacokinetic parameters | 3 |
|  |  | In vivo processes are unclear, or no pharmacokinetic studies are available | 1 |
|  | Pharmaceutical formulation and administration (multiple choice)  (12 points) | Main ingredients and excipients (all specify 1; one specify 0.5) | 1 |
|  |  | Specification and packaging (all appropriate for clinical use/dose adjustment 1; one appropriate 0.5) | 1 |
|  |  | Dosage forms (oral/inhalation/topical formulations 1; subcutaneous/intramuscular injections 0.5) | 1 |
|  |  | The dose administered (fixed dose 2; dose to be adjusted during use 1.5; dose based on body mass or body surface area 1) | 2 |
|  |  | Frequency of administration (once every 4 weeks or more 2; once every 1 to 4 weeks 1.8; once every 1 day to 1 week 1.5; twice a day 1; three or more times a day 0.5) | 2 |
|  |  | Medication administration time (No need to administer at a specific time 2; Need to administer at a specific time 1) | 2 |
|  |  | Ease of use (self- administration without assistance 3; with help or training 2; administered by medical personnel 1) | 3 |
|  | Storage  conditions  (multiple choice)  (3points) | Storage at room temperature | 2 |
|  |  | Storage in the shade | 1.5 |
|  |  | Refrigerated/frozen storage | 1 |
|  |  | No need for shade/light protection | 1 |
|  | Drug shelf life (2 points) | ≥36 months | 2 |
|  |  | ≥24 months, <36 months | 1.5 |
|  |  | ≥12 person-months, <24 months | 1 |
|  |  | <12 months | 0.5 |
| **Economy (10 points)** | Same Generic Name Drug (3 points) | Daily treatment cost of the lowest-priced drug is 3 points; Evaluation drug score = Lowest daily treatment cost /average daily treatment cost of the evaluated drug × 3 | 3 |
|  | Alternative drugs with the main indications (7 points) | Daily treatment cost of the lowest-priced drug is 7 points; Evaluation drug score = Lowest daily treatment cost / average daily treatment cost of the evaluated drug × 7 | 7 |
| **Other attributes (8 points)** | National Health Insurance (3 points) | National medical insurance category A, no payment limitations | 3 |
|  |  | National medical insurance category A with payment limitations | 2.5 |
|  |  | National medical insurance category B, no payment limitations | 2 |
|  |  | National medical insurance category B with payment limitations | 1.5 |
|  |  | Drugs included in the commercial health insurance innovative medicine list | 1 |
|  | National Essential Drugs (1 points) | National essential drugs without Δ requirement | 1 |
|  |  | National essential drugs with Δ Requirements | 0.5 |
|  | National Centralized Drug Procurement (1 point) | Selected drugs for centralized national procurement | 1 |
|  | Originator/Reference/Consistency Evaluation (1 point) | Drug of origin/reference drug | 1 |
|  |  | Generic drugs through consistency evaluation | 0.5 |
|  | Manufacturer ranking (1 point) | The world’s top 50 pharmaceutical manufacturers in terms of sales volume / Top 100 Pharmaceutical Industry published by Ministry of Industry and Information Technology | 1 |
|  | Global Usage (1 point) | Available in China, the United States, Europe, Japan | 1 |
|  |  | Domestic and international sales | 0.5 |

**Supplementary Table 2 Search strategies for PubMed**

| **English Search terms** | |
| --- | --- |
| 1 | ("Kidney Failure, Chronic"[Mesh] AND "Anemia"[Mesh]) OR "renal anemia"[Title/Abstract] OR "anemia of renal disease"[Title/Abstract] OR "anemia of chronic kidney disease"[Title/Abstract] OR "anemia in chronic kidney disease"[Title/Abstract] OR "anemia of CKD"[Title/Abstract] OR "CKD-associated anemia"[Title/Abstract] OR "anemia in hemodialysis patients"[Title/Abstract] OR "anemia in dialysis patients"[Title/Abstract] OR "anemia of end-stage renal disease"[Title/Abstract] OR "uremic anemia"[Title/Abstract] |
| 2 | "Erythropoietin"[Mesh] OR "erythropoiesis-stimulating agents"[Title/Abstract] OR "ESAs"[Title/Abstract] OR "long-acting erythropoietin"[Title/Abstract] OR "long-acting ESA"[Title/Abstract] OR "long-acting erythropoiesis-stimulating agent"[Title/Abstract] OR "darbepoetin alfa"[Supplementary Concept] OR "darbepoetin alfa"[Title/Abstract] OR "darbepoetin"[Title/Abstract] OR "methoxy polyethylene glycol-epoetin beta"[Supplementary Concept] OR "methoxy polyethylene glycol-epoetin beta"[Title/Abstract] OR "continuous erythropoietin receptor activator"[Title/Abstract] OR "CERA"[Title/Abstract] OR "peginesatide"[Supplementary Concept] OR "peginesatide"[Title/Abstract] OR "Omontys"[Title/Abstract] |
| 3 | (("randomized controlled trial"[Publication Type] OR "controlled clinical trial"[MeSH Terms] OR "randomized controlled trials"[All Fields]) OR ("meta-analysis"[Publication Type] OR "meta-analysis as topic"[MeSH Terms] OR "meta-analysis"[All Fields])) |
| 4 | 1 AND 2 AND 3 |
